# Occupational Exposure to Polycyclic Aromatic Hydrocarbons and Elevated Cancer Incidence in Firefighters

**DOI:** 10.1038/s41598-018-20616-6

**Published:** 2018-02-06

**Authors:** Anna A. Stec, Kathryn E. Dickens, Marielle Salden, Fiona E. Hewitt, Damian P. Watts, Philip E. Houldsworth, Francis L. Martin

**Affiliations:** 1 Centre for Fire and Hazards Sciences, School of Physical Sciences and Computing, University of Central Lancashire, Preston, PR1 2HE UK; 2 Hampshire Fire and Rescue, Leigh Road, Eastleigh, SO50 9SJ UK; 3 School of Forensic and Applied Sciences, University of Central Lancashire, Preston, PR1 2HE UK; 4 School of Pharmacy and Biosciences, University of Central Lancashire, Preston, PR1 2HE UK

## Abstract

Cancer incidence appears to be higher amongst firefighters compared to the general population. Given that many cancers have an environmental component, their occupational exposure to products of carbon combustion such as polycyclic aromatic hydrocarbons (PAHs) is of concern. This is the first UK study identifying firefighters exposure to PAH carcinogens. Wipe samples were collected from skin (jaw, neck, hands), personal protective equipment of firefighters, and work environment (offices, fire stations and engines) in two UK Fire and Rescue Service Stations. Levels of 16 US Environmental Protection Agency (EPA) PAHs were quantified together with more potent carcinogens: 7,12-dimethylbenzo[a]anthracene, and 3-methylcholanthrene (3-MCA) (12 months post-initial testing). Cancer slope factors, used to estimate cancer risk, indicate a markedly elevated risk. PAH carcinogens including benzo[a]pyrene (B[a]P), 3-MCA, and 7,12-dimethylbenz[a]anthracene PAHs were determined on body surfaces (e.g., hands, throat), on PPE including helmets and clothing, and on work surfaces. The main exposure route would appear to be via skin absorption. These results suggest an urgent need to monitor exposures to firefighters in their occupational setting and conduct long-term follow-up regarding their health status.

## Introduction

In most states in Canada and in the majority of US states, presumptive legislation recognizes certain cancers as occupational diseases amongst firefighters. For example in British Columbia, the following cancers are now recognized as firefighter occupational diseases: brain, bladder, colorectal, kidney, ureter, testicular, lung, esophageal, non-Hodgkin’s lymphoma, leukemia, breast, prostate and multiple myeloma.

The main consensus of such studies show that the incidence of cancer is higher amongst firefighters than the populations they serve despite the health benefits of their superior physical fitness^1–3^. A meta-analysis of 32 studies published in 2006 showed increasing summary risk estimates for multiple myeloma, non-Hodgkin’s lymphoma, prostate cancer and testicular cancer with eight other cancers having a possible association with firefighting^1^. A later pooled cohort study, from National Institute for Occupational Safety and Health (NIOSH), of 29,993 US firefighters with 6.9% cancer mortality with a further 13.5% cancer incidence, showed clear associations with digestive and respiratory cancers, alongside excess mesotheliomas (associated with asbestos exposure)^2^. A similar study of 16,422 Nordic firefighters showed an increased risk of prostate cancer, skin melanoma and non-melanoma skin cancer, multiple myeloma and adenocarcinoma of the lung, as well as mesothelioma^4^. Although melanomas are frequently associated with exposure to ultraviolet radiation, they have also been found on the unexposed skin of petrochemical refinery workers, demonstrating a significant positive association between melanoma and exposure to benzene and polycyclic aromatic hydrocarbons (PAHs)^5^.

Smoke “diving’’ simulations show an increase and a delay in the urinary excretion of PAH metabolites in urine (1-hydroxypyrene (1-OHP) and 1-hydroxynaphthalene) and established the importance of the multiple exposure pathways of inhalation and dermal absorption of PAHs^6,7^ Studies by NIOSH showed that even a self-contained breathing apparatus (SCBA) worn and evaluated as fully operational is contaminated within 25 minutes of use in firefighting situations^8^. These results signify the build-up of toxicants of firefighters’ ensembles with repeated use resulting in increased toxic exposure to firefighters from just their ensemble^9^. A general PAH increase in the post-exposure of firefighter training was identified in other studies, with the highest increase on the SCBA^7,10^, PAHs were identified on the inner surfaces of the PPE even when firefighters wore a full set of PPE^11^. The neck is to be found the primary site of dermal exposure and the PPE hood only provides minimal protection^8^. When firefighters remove their PPE, dermal and inhalation exposure still exists through contact with contaminants. In a study of coke oven workers, it was estimated that 95% of PAHs enters through the skin^12^. A publication by Stull additionally shows that the tear resistance and seam strength decrease with application of laundering and that the decontamination efficiency markedly varies with the chemical and that 100% of the decontamination is not removed^13^.

Many PAH studies have found potential links to certain cancers, but have not explicitly proven that these agents are causative, as there is insufficient epidemiological/experimental data on individual PAHs and mixtures thereof. Most PAHs are not only pro-carcinogens, but also are listed as genotoxic and mutagenic. The carcinogenicity of PAHs, as well as their Toxic Equivalency Factor (TEF), is related to one of the most significant of the pyrogenic carcinogens, benzo[a]pyrene (B[a]P). However, recent environmental studies identified PAHs of considerably higher toxicity than the US Environmental Protection Agency (EPA) 16 priority PAHs^14^. From a regulatory perspective many of these have not been studied in sufficient detail with respect to frequency of occurrence in the environment and to their toxicity to different organisms to justify their inclusion in routine measurements^12^. For example, 3-methylcholanthrene (3-MCA) possesses a carcinogenic potential approximately 5.7-fold that of B[a]P, meaning that low concentrations of them may contribute significantly to the toxic effects of exposure. Alkyl derivatives of PAHs, for example 7,12-dimethylbenzo[a]anthracene, have a 20-fold higher TEF than its parent compound and twice that of B[a]P^14^. Selected PAHs studied in this work, their exposure limits (TEFs, inhalation unit risks) together with their toxic effect on humans is presented in Supplemental Material 1^14–16^.

The aim of this study was to conclusively demonstrate the elevated occupational exposure of firefighters to individual carcinogenic PAHs.

## Methods

These are the first UK studies on firefighters and their occupational environment to identify possible contaminants that may have an effect on their health. Skin, clothing and other sample locations were identified based on previously published resources^6,13,17–21^.

The focus of these studies was on 16 standard EPA PAHs. However, herein only those listed as group 1 or 2 (carcinogens or probably/possibly carcinogenic to human) according International Agency for Research on Cancer (IARC) and US Environmental Protection Agency (EPA) are presented. All samples were re-run 12 months after initial sampling for three other PAHs: 3-MCA, 7,12-dimethylbenzo[a]anthracene and dibenzo(a,e)pyrene, which may be more harmful than B[a]P.

Studies were carried in two UK Fire and Rescue Service Stations (identified as Station 1 and Station 2), on the southern coast of England. Wipe samples were collected from the skin, the personal protective equipment (PPE) of firefighters, and selected work surfaces. In addition, airborne PAHs were sampled and trapped on sorption tubes (XAD) from the general office and PPE storage area of each of the stations. In Station 1, PPE was stored in a separate room and in Station 2, PPE was stored in the appliance bay. The actual storage method of helmets and gloves was observed, and the PAH deposition was investigated as a function of the different storage habits, together with the effectiveness of the cleaning methods of the firefighters’ protective clothing. All data generated including sampling details and analytical methods are provided in Supplemental Material 2 and 3.

Ethical approval was obtained from an Employees’ Committee with line management authorisation serving as the local Institutional Review Board. This study was conducted according to the principles of the Declaration of Helsinki and all other applicable national or local laws and regulations. All participants gave written informed consent before any protocol-specific procedure was performed.

### Sample Collection

#### Skin and clothing sample collections

Skin and clothing samples were collected pre- and post- training exercise for firefighters, which occurred in a single 3 m × 9 m × 2.4 m shipping container, in which a 2 m × 3 m piece of oriented strand board (OSB) was burnt. Firefighter subjects were randomly chosen, 3 trainees out of the 8 firefighters and 1 instructor out of 3. The instructor was very close to the container opening during the entire event, whilst the trainee firefighters entered the container twice, for approximately 5 minutes; one positioned at the front, controlling the firefighting branch, and the other at the rear controlling length of the hose. Both firefighters are exposed to heat and smoke possibly in equal measures.

Each exercise lasted approximately 60 minutes. Wipe samples were taken prior and approximately 10 minutes after the exercise from different skin areas (back and front of neck, jawline and hands) and from PPE (exterior of gloves, jacket zipper cover, shoulder of firefighter tunic, flash hood and exterior of SCBA mask). The firefighters also completed a questionnaire about both their fire role and other activities, to identify other potential sources of PAH exposure(s), presented in Supplemental Material 4.

#### Fire Stations

Wipe and gas samples were taken from each office and from fire engines at each fire station. Both stations are cleaned regularly (i.e., daily) by cleaning staff. In Station 1, wipe samples were taken in the PPE storage room and the general office where atmospheric sampling was also undertaken. In Station 2, wipe and atmospheric samples were taken from the general office and the appliance bay which houses the fire engines and the PPE store. Doors and windows to the bay were generally closed during sampling.

To account for any variation within the rooms, three samples were taken from the offices of each station from different locations within the rooms (and are added and presented as one bar). Since office staff are likely to touch a larger surface area than fire crews in vehicles etc. Wipe samples were taken from the wall on immediate entry to the room, in the middle of the room, and from the far wall. In addition to the fire offices, samples were collected in the PPE storage room in Station 1 and the PPE storage space in the appliance bay of Station 2. Wipe and atmospheric samples were collected from the top shelf (for helmets) and the bottom shelf (for boots) added and presented, since contact with both surfaces seems likely to occur. Windows and doors were kept closed during atmospheric sampling, but were opened occasionally during sampling by firefighters working in that area.

#### Fire Engines

Wipe and atmospheric samples from different fire engines (FE1-FE3) based at Station 1 were taken a few days prior to, and after a fire at a derelict former cinema. Wipe samples were taken from the electronic controller (bodyguard, Drager Bodyguard 7000) of the SCBA located on the right side of the vehicle, the back-right door and the console next to the driver’s seat. These locations were selected as they have a large surface area that is mostly left undisturbed or cleaned. Atmospheric samples were taken overnight from the crew side of the dividing partition (between the crew cab and the driver’s compartment) from the three fire engines that attended the fire at the cinema. The doors of the fire engines were closed during sampling and any PPE that didn’t belong in the cab was removed, in order to avoid cross-contamination. Previous studies carried out in appliance bays have suggested that the diesel emissions from the exhaust system of the engine itself can increase the PAHs levels; however in both cases, the fire appliance bays had an exhaust system designed to mitigate this.

#### Sample collection from used and cleaned gear

Firefighters are usually provided with two sets of PPE and it is cleaned in three different ways. Firefighters have two complete sets of PPE excluding helmet and boots (tunic, trousers, gloves, hood). The tunic and leggings will be sent to the manufacturer and the other factors are dependent on the firefighter decision when to clean on station.

- Surface dirt brushed off - if the PPE has surface dirt then a stiff brush might be used to clean it especially if it’s only a small area. For example, the bottom of the trousers might have some surface dirt on it after turnover (firefighter may not have been in the fire but is making sure the fire is fully out by moving objects). If that’s the only bit of dirt they will brush it off.
- Washed in a “domestic” washing machine at 40 °C using normal detergent powder- if the PPE has lots of dirt and smells then it will be washed on station by the firefighter. The wash cycle is normally 45 minutes. The PPE will then be hung to dry in a room (drying room) that is kept hot. Lots of PPE will generally be in there and also personal kit such as towels and clothes.
- Every six months two sets tunic and trousers are sent away to the manufacturer as part of a service package. The PPE will be washed, inspected for damage and repaired. The PPE is returned if it’s good or kept if it fails, when a new set is issued. This is the set that was tested after washing. It is only the tunic and trousers that are done this way. The gloves, boots and hood are all cleaned at station.

All instances of cleaning are recorded. The flash hood is not a part of this process, and is only cleaned on-station in the washing machine.

Wipe samples were taken from four sets of PPE in the storage room at Station 1, from the inside the headband of each helmet and the corresponding sets of gloves, two of which were stored inside the helmets in the helmet bag, one pair that was stored in the boots and one pair that was stored in the pocket of the jacket, following the actual storage practice used by each firefighter. In addition, wipe samples were taken from the shoulders and zip flap of two jackets and from the lower sections of two pairs of trousers, of two sets of PPE that have recently returned after being cleaned according to the manufacturer’s process.

## Results

### Skin and clothing samples

**Figure 1:**
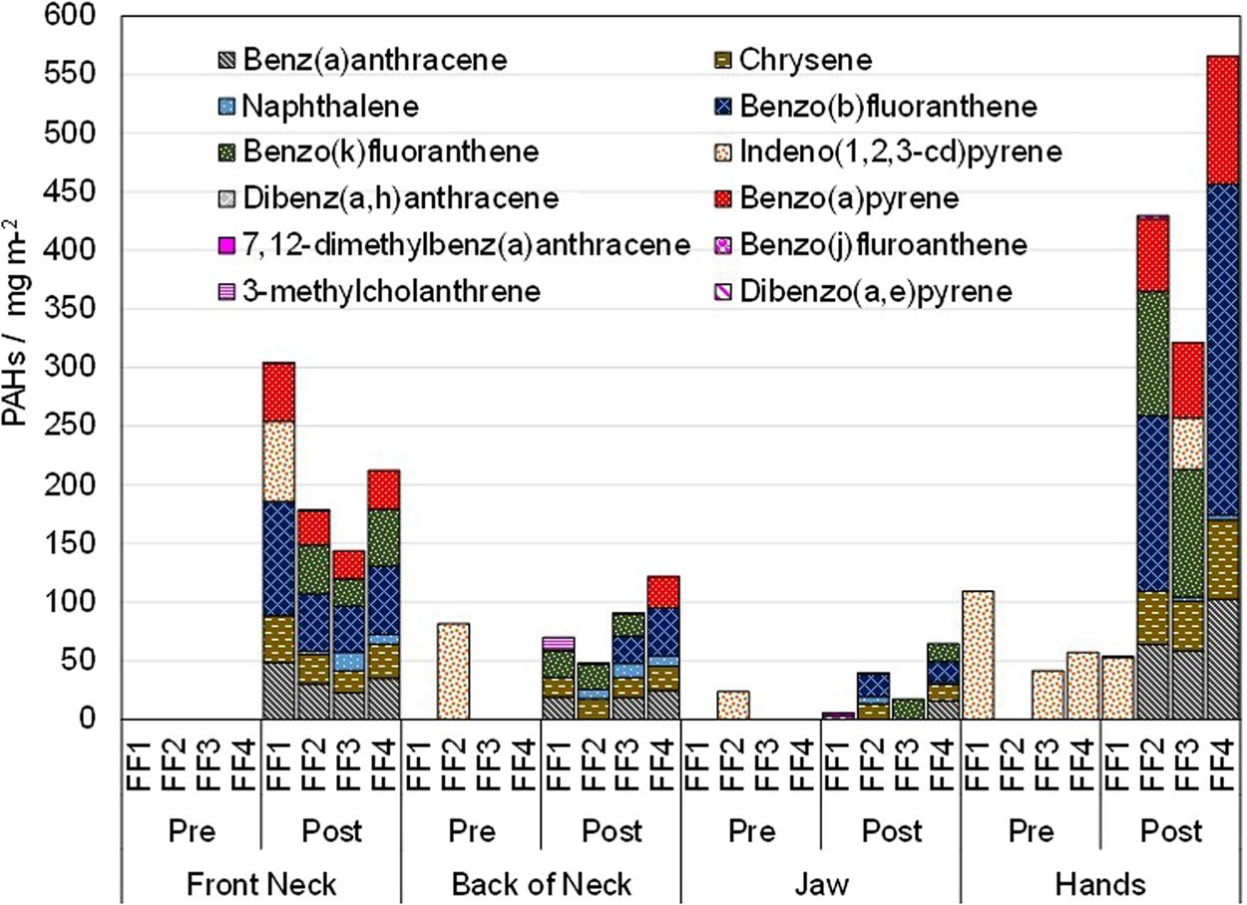
PAHs concentration in skin wipe samples, pre- and post-exposure, of four firefighters attending the training.

PAH concentrations in skin wipe samples, pre- (pre) and post-exposure (post), of an instructor (FF1) and three trainees (FF2-FF4), attending the training are presented in Fig. 1. A significant increase in concentration for the majority of the studied PAHs is noticeable on the four sampled locations, especially the hands. The samples from the instructor show changes in post-exposure samples for the neck, which may be due to contaminated clothing. A similar profile is observed for hand samples for most of the firefighters studied. Post-training, B[a]P was found on hands and throat for almost all the firefighters, on the neck of FF4, but not detected on any of the samples collected from the jaw.

**Figure 2:**
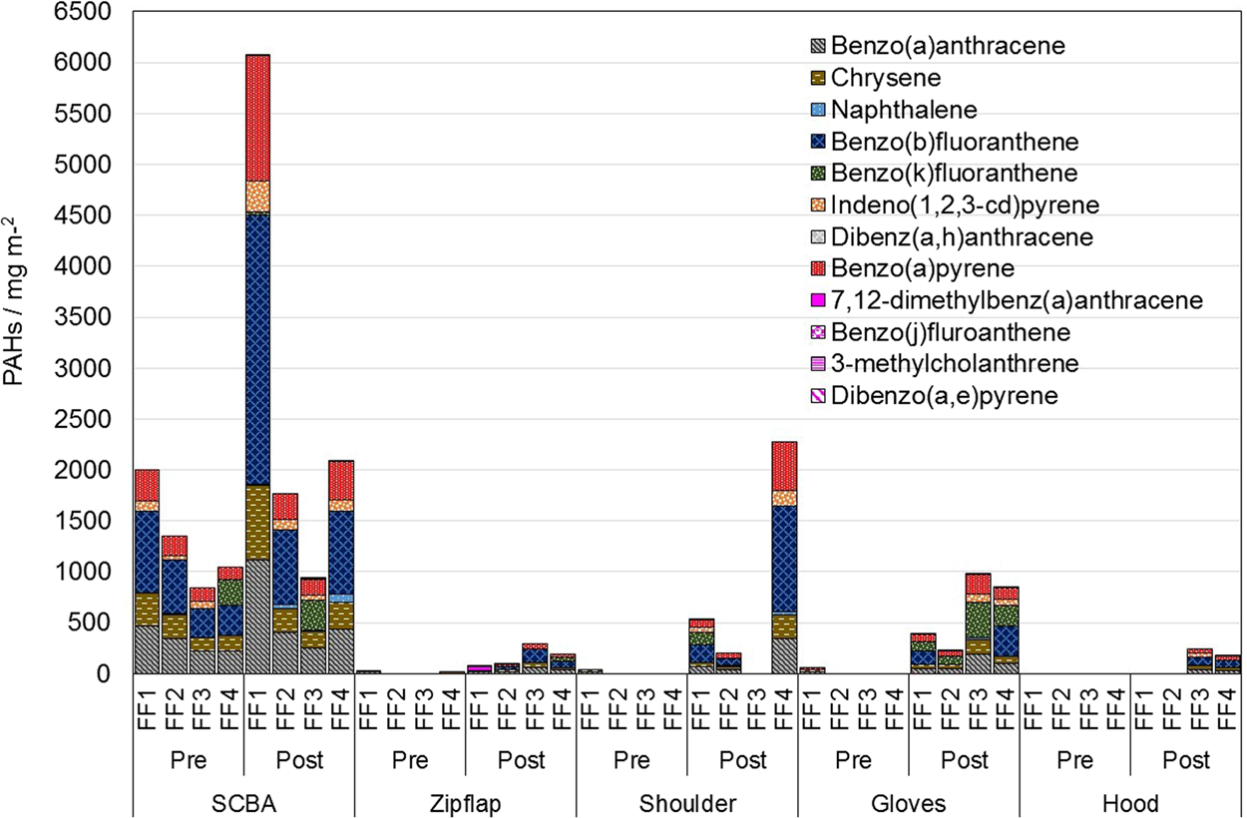
Total PAHs concentration found in PPE samples from firefighters attending training, taken pre- and post-exposure from five locations.

The results of the quantitative analysis of the PPE wipe samples from the firefighter training are presented in Fig. 2. The PAH concentrations in pre-exposure wipe samples taken from the outside of the SCBA masks was visibly higher than the other pre-exposure samples taken from the shoulders, zipper covers, hoods and gloves. The SCBA used by the firefighters during training events is not part of their own PPE, but provided by the training facility and used for all training sessions. Between sessions they are cleaned with soap and water. This, together with the high concentrations of PAHs found in the samples taken from the masks, shows that the cleaning process is not sufficient.

A similar trend is seen for all the other obtained post-exposure samples with a significant increase in PAHs concentrations. B[a]P was identified on the SCBA and gloves of every firefighter tested, and almost on all the zip flap, shoulder and hood samples. Very high levels on SCBA of FF1 and FF4 and on the shoulder of FF4’s PPE and gloves of FF3 and FF4 are a cause for concern. B[a]P concentrations for SCBA of FF1 itself has more than 1000 mg/m^2^. Without robust protocols to mitigate such exposures, it is likely to significantly increase exposure to carcinogenic PAHs.

### PPE clothing

**Figure 3:**
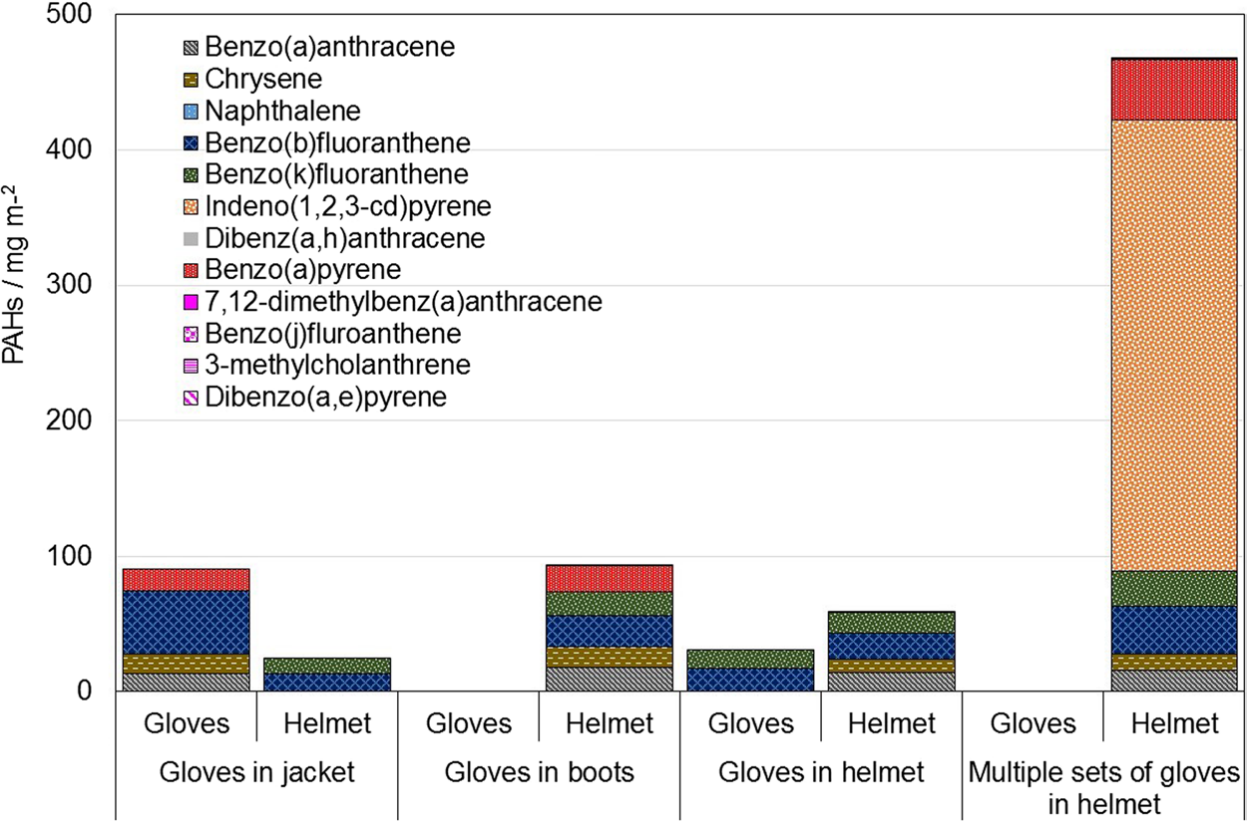
Sampling from used and cleaned PPE.

The results of the quantitative PAHs analysis of PPE clothing, stored in different configurations, are presented in Fig. 3. It is unknown when the PPE was last used and if any of them had recently been worn into a fire site. The recent history of the PPE is unknown, but gives a snapshot of likely PAH exposures in the firefighter’s working environment. In the helmets, the highest concentration of PAHs was found when it had been used to store several pairs of gloves, whereas the lowest concentration was found when the gloves had been stored in the jacket. For the gloves, the pattern was reversed with the highest concentration occurring when they were stored in the jacket and the lowest when they were stored as multiples in the helmet. The trend suggests there is a possibility that storing gloves in helmets may lead to transfer of contaminants from the gloves to the inside of the helmet. This may arise from the presence of coating of an oily/greasy layer from human hair on the inside of the helmet. At first sight this could be attributed to volatile equilibriums, but the boiling points of the larger PAHs are too high, so it seems more likely to result from physical contact of gloves and helmet.

In addition, wipe samples taken from three locations (shoulder, zip flap and trousers) on two professionally cleaned PPE suits “using standard detergent solution” were also obtained. Only acenaphthylene, pyrene, anthracene and fluoranthene were identified (non-carcinogenic) and in relatively small concentrations. The low levels of PAHs are in accordance with the report by Stull in which it is stated that laundering processes have an overall high decontamination efficiency, which is also dependent on the compounds present during decontamination, but this process is not 100% efficient^13^.

### Fire Stations and Engines

**Figure 4:**
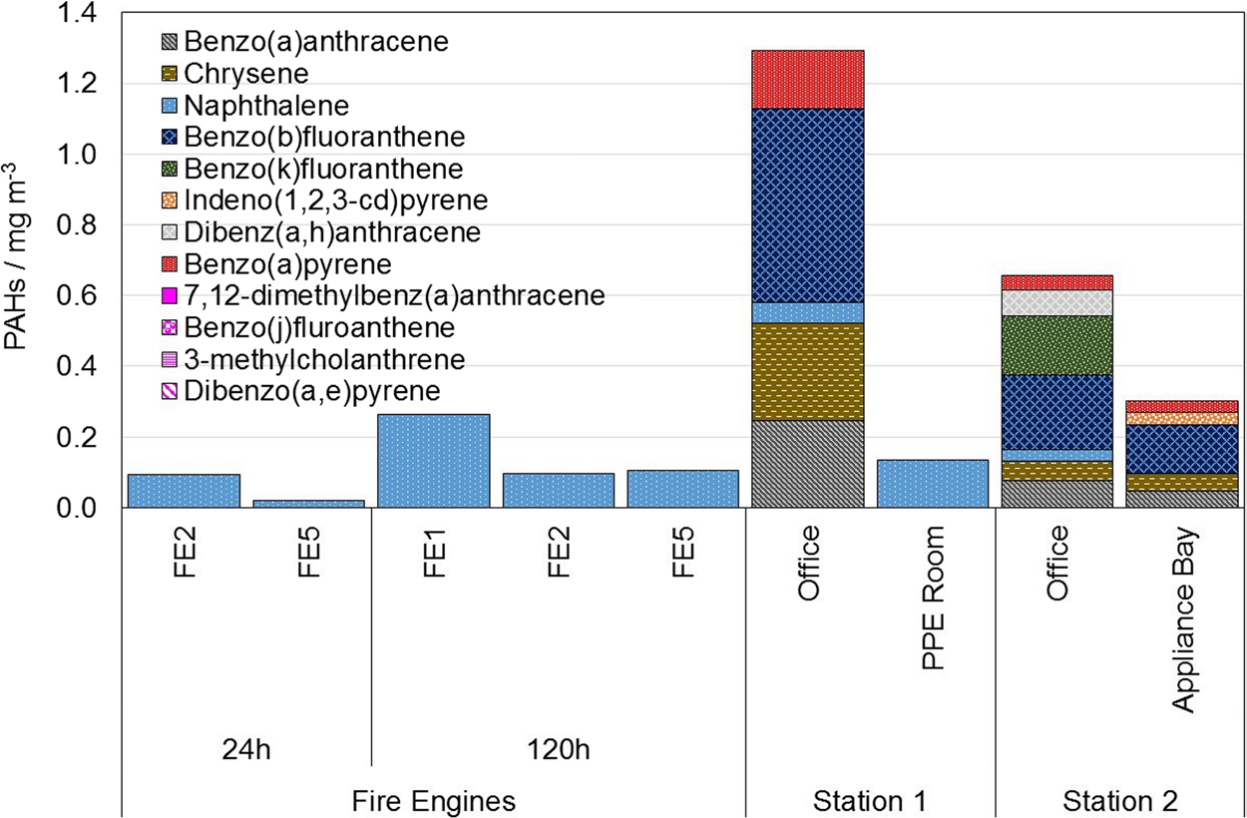
PAHs summary for fire engines and stations from the XAD filters.

**Figure 5:**
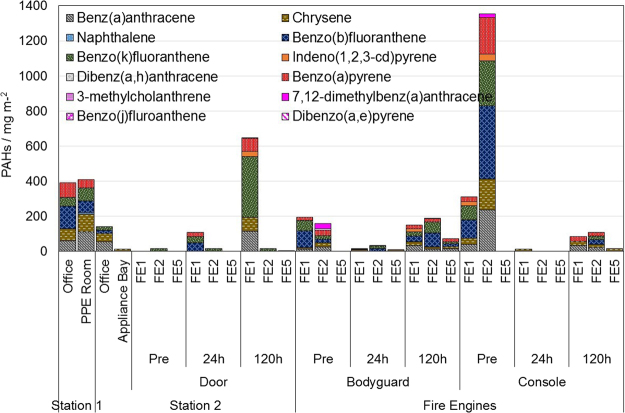
PAHs summary for fire engines and stations collected from the wipe samples.

The results of the quantitative analysis of the gas and wipe samples from both of the stations are presented in Figs 4 and 5. For fire engines (Fig. 4), only naphthalene was found. B[a]P was identified within all XAD samples for the offices, as well as from the appliance bay of the second station. Naphthalene, chrysene, benzo(a)anthracene, and benzo(b)fluoranthene were identified for all locations.

Figure 5 shows the PAH concentration for the two fire engine samples collected prior to, and one, five days after the fire in the disused cinema. There is an increase in PAH concentration of the wipe samples with time since exposure, which is independent of the sampling location. B[a]P and class 2 carcinogens are found in all sampled locations either prior to, or after exposure or both. This shows that the levels of air sampled gaseous phase PAHs and the levels of wipe sampled condensed phase PAHs are not in a constant proportion.

## Discussion

The purpose of this study was to identify the occupational exposure of firefighters to PAHs, and to establish whether particular practices may adversely affect their health. Sixteen EPA priority PAHs plus the more recently recognized carcinogenic 7,12-dimethylbenz(a)anthracene, 3-MCA and dibenzo(a,e)pyrene have been quantified from wipe samples from firefighters’ skin, personal protective equipment and working environment and from gas sampling in the fire stations and in fire engines. It is interesting to surmise that such elevated exposures may underlie elevated cancer incidence in firefighters.

7,12-Dimethylbenz(a)anthracene was not identified on the skin samples, but was found on the wipe samples from clothing (zipflap) and fire engines before and after the exposure (console and bodyguard). 3-MCA was identified on the skin samples (neck, jaw), SCBA and pre-post exposure samples from the door and bodyguard of the fire engines.

**Figure 6:**
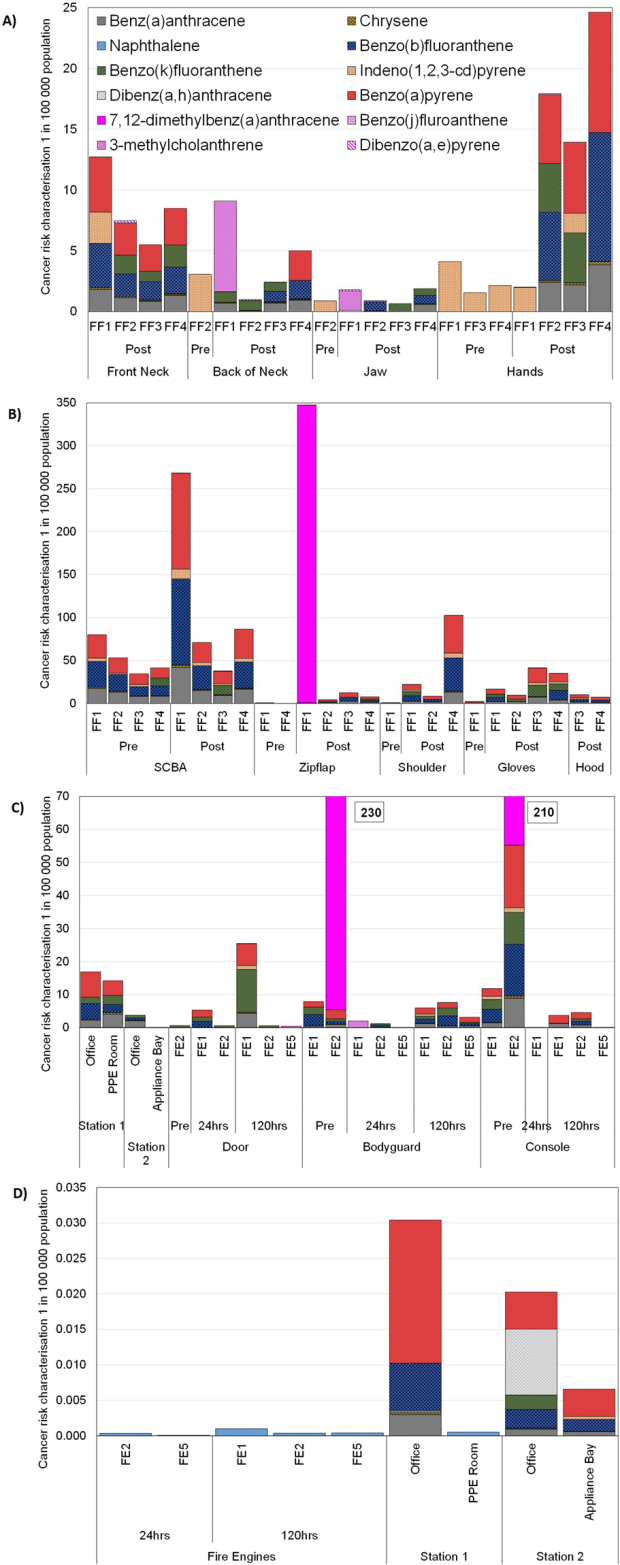
Cancer risk characterization (1 in 100 000 population) via skin absorption for (**A**) skin, (**B**) clothing and (**C**) fire engines and fire offices and (**D**) via inhalation route for fire engines and fire offices.

Cancer slope factors (CSF) are used to estimate the risk of cancer from a lifetime exposure to an agent by ingestion or inhalation. Based on California’s Proposition 65 Law, results from the skin and clothing samples were used to evaluate the risk assessment, via dermal exposure, for cancer endpoints^16,22^, As explained in the EPA Guidelines for Carcinogen Risk Assessment, defined risk factor is estimated for 1 in 100,000 cases of cancer per population. Health Canada Relative Dermal Absorption Factors concluded that PAHs have a dermal absorption of 20%. Exposure scenarios assume that the average firefighter would wear the turnout gear 2 days a week for 50 weeks per year over the span of their career, which considers 40-years for worst case and the lifetime to be 70 years^23^. For clothing samples, the worst-case approach considered that 1 m^2^ of the garment transferred the extractable amount to the skin^24–26^. The risk characterization for the cancer (1 in 100 000 population) is shown in Fig. 6.

Dermal absorption rates via skin in scalp, axilia, forehead behind ear, jaw is much faster than top or the palm of the hand as thicker skin can offer greater resistant to toxicants than those areas with thinner skin. In almost all cases high or very high quantities of PAHs were identified. Based on a cancer risk factor of 1 in 100 000, it was found that the highest risk is obtained mainly from the dermal exposure. Up to 350 firefighters can develop cancer from PAHs concentrations identified on the clothing. Up to 230 and 210 firefighters may develop cancer from PAHs found on the fire engines bodyguard and console respectively. Higher Risk Characterization Ratio of 25 was identified from PAHs identified on firefighters hands. There is negligible risk identified from the gas sampling either in fire engines or fire stations. B[a]P levels are in most of the cases much higher than those established by the Californian Proposition. 7,12-Dimethylbenz(a)anthracene and 3-MCA also exceeds limits for the samples that identified those PAHs.

These results emphasize also the need to determine the exposure levels of other combustion-generated contaminants. In addition, it will be critical to determine the effects of such exposures either singly or as mixtures along with the modifying effects of different contaminant constituents in a mixture on each other^27–29^. Elucidating effects on representative target cells will play a critical role in assessing risk. Further studies could look for chromosomal aberrations in erythrocytes or bulky chemical-DNA adducts in lymphocytes or effects (e.g., epigenetic) on circulating free DNA^30^.

Long-term and detailed follow-up studies are critical in order to capture individual PAHs exposures to be responsible for elevated cancer incidence in firefighters. In line with such studies, it is important to track and monitor as many active firefighters as possible in order to generate a robust risk paradigm.

## Electronic supplementary material

Supplemental Material
