# Supplementary material for: Occupational Exposure to Polycyclic Aromatic Hydrocarbons and Elevated Cancer Incidence in Firefighters
Source: Sci Rep. 2018 Feb 6;8:2476. doi: 10.1038/s41598-018-20616-6 (PMC5802792; doi:10.1038/s41598-018-20616-6)
Supplement: Supplementary file 1 — Supplemental Material [file 41598_2018_20616_MOESM1_ESM.pdf]

Supplemental Material

Occupational Polycyclic Aromatic Hydrocarbons and Elevated Cancer Incidence in Firefighters

Anna A Stec, Kathryn E Dickens, Marielle Salden, Fiona E Hewitt, Damian P Watts, Philip E Houldsworth, Francis L Martin

Supplemental Material 1

Table S1: PAH exposure limits and their toxic effect on a human Achten and Andersson 2015; Agency for Toxic Substances and Disease Registry 2017; IARC 2010; Office of Environmental Health Hazard Assessment (OEHHA) 1992, 2011, 2017; US EPA 1988, 1990).

	**Toxicity Mechanism**	**TEF**	**IARC**	**EPA**	**Inhalation Unit risk (mg/m^3)**	**NSRL (mg per day)**	**Inhalation Slope Factor (mg/kg per day)**	**Oral Slope Factor (mg/kg per day)**
**Benzo(a)pyrene** 50-32-8	Tumours at several different tissue sites respiratory and digestive system, forestomach and oesophagus, lung, mammary gland and skin	1	1	B2	0.0011	0.06	3.9	2.9
**Dibenz(ah)anthracene** 53-70-3	Oral or dermal and injection leading to cancer of the lung (adenomatosis or alveologenic carcinoma) and mammary gland (carcinoma), benign or	10	2A	B2	0.0012	0.2	4.1	4.1

	malignant tumours of the forestomach (squamous-cell papilloma or carcinoma), and tumours of the blood vessels (hemangioendothe glioma)							
**Naphthalene**<br>91-20-3	Inhalation led to induced inflammation of the nose and lung, metaplasia of the olfactory epithelium, and hyperplasia of the nasal respiratory epithelium. Increased the incidence of benign lung tumours (alveolar/bronchiolar adenomas) a malignant lung tumour (alveolar/bronchiolar carcinoma) also laryngeal cancer cases and colorectal carcinoma.	0.001	2B	C	0.000034	5.8	0.12	0.12

**Benz(a)anthracene** 56-55-3	Benign or malignant lung tumours (adenoma or adenocarcinoma), liver cancer (hepatocellular carcinoma), skin tumours after application to the skin, sarcoma at the injection site and urinary-bladder cancer (carcinoma) after implantation in the bladder.	0.2	2B	B2	0.00011	0.033 (oral)	0.39	1.2
**Chrysene** 218-01-9	Carcinomas and malignant lymphoma was identified after intraperitoneal injection and skin carcinomas following dermal exposure.	0.1	2B	B2	0.000011	0.35 (oral)	0.039	0.12
**Benzo(b)fluoranthene** 205-99-2	Dermal application caused skin tumours, and subcutaneous injection cancer at the injection site (sarcoma). Exposure led to	0.8	2B	B2	0.00011	0.096 (oral)	0.39	1.2

	lung cancer (carcinoma) benign lung tumours (adenoma), and malignant liver tumours (hepatocellular adenoma)							
**Benzo(k)fluoranthene** 207-08-9	Tumours at two different tissue sites, and by two different routes of exposure: lung cancer (squamous-cell carcinoma) and cancer at the injection site (sarcoma)	0.2	2B	B2	0.00011	X	0.39	1.2
**Indeno(1,2,3-dc)pyrene** 193-39-5	Dermal exposure caused benign and malignant skin tumours (papilloma and carcinoma) and subcutaneous injection caused cancer at the injection site (sarcoma), lung cancer (carcinoma)	0.1	2B	B2	0.00011	X	0.39	1.2
**Benzo(j)fluoranthene** 205-82-3	Dermal exposure caused benign or malignant skin	0.2	2B	X	0.00011	0.011 (oral)	0.39	1.2

	tumours (papilloma or carcinoma). Exposure led to lung cancer (squamous-cell carcinoma) benign and malignant lung tumours (alveolar/bronchiolar adenoma and carcinoma) and liver tumours (hepatocellular adenoma or carcinoma)							
**7,12-dimethylbenz(a)anthracene** 57-97-6	When applied to skin can be both a carcinogen and as a tumor initiatior. Sarcomas after injection and lung tumors after intratracheal instillation. Mammary cancer or fibriadebinas, carcinomas, adenocarcinomas and leukemia, tumors in the mammary gloand, ovaries,	20	X	X	0.071	0.003	250	250

	pancreas by direct application to the target organ.							
**3-methylcholanthrene** 56-49-5	Liver and lung tumors, neoplastic changes in lungs, skin and mammary gland, carcinogenesis (proliferation), dystrophy and necrosis were all identified.	5.7	X	X	0.0063	0.03	22	22
**Dibenzo(a,e)pyrene** 192-65-4	Very limited data avialable	1.0	3	B2	0.0011	X	3.9	12

**IARC Classification:**	**US EPA Classification:**
Group 1 Carcinogenic to humans	Group A - Carcinogenic to Humans:
Group 2A Probably carcinogenic to humans	Group B - Probably Carcinogenic to Humans
Group 2B Possibly carcinogenic to humans	Group C - Possibly Carcinogenic to Humans
Group 3 Not classifiable as to its carcinogenicity to humans	

Supplemental Material 2

Sampling details

Samples were collected from a firefighter training exercise which occurred in a single 3 m x 9 m x 2.4 m shipping container, in which a 2 m x 3 m piece of oriented strand board (OSB) was burnt. Firefighter subjects were randomly chosen, 3 trainees out of the 8 firefighters and 1 instructor out of 3. The instructor was very close to the container opening during the entire event, whilst the trainee firefighters entered the container twice, for approximately 5 minutes; one positioned at the front, controlling the firefighting branch, and the other at the rear controlling length of the hose. Both firefighters are exposed to heat and smoke possibly in equal measures.

Skin and PPE wipe samples taken using pre-made, individually wrapped Cutisoft wipes from MediSupplies (Bournemouth, UK) (6 x 3 cm, pre-impregnated with 70% v/v isopropyl alcohol). Sample collectors wore nitrile gloves and gloves changed between each collection site to avoid cross contamination.

Skin wipe samples were taken from: the back of the neck (horizontally just above the collar), palms of the hands (from wrist to fingertips), throat and jawline (horizontally just above the collar).

Clothing wipe samples were taken from the shoulders of jacket (taken from seam to elbow), jacket zipper cover (top of collar to height of lower front pocket), fingers of gloves (base of finger to tips), jacket hood, base of hood (that has contact with neck of firefighter).

Equipment wipe samples were taken from breathing apparatus mask (outside surface around filter).

Wipe samples, using 10x11cm Kimtech tissues impregnated with 2.0mL isopropyl alcohol, were taken from the station offices, doors and ridge surfaces, fire engines inside of fire doors and front of bodyguard along the edge of the console. Additionally wipe samples were taken from cleaned and laundered clothing (shoulders of jacket, and jacket zipper cover, trousers).

Gas samples were collected using XAD-2 tubes from SKC Ltd. The XAD filter samples were collected for 6 hours using portable air pumps set at 1.0 L/min. Gas samples were collected from both of the fire stations (Station 1 and 2): general office and PPE room in Station 1 and general office and appliance bay in Station 2. Additional gas samples were collected from three fire engines from area between crew cab and drivers compartment.

XAD tubes were sealed with hats and wrapped in aluminum foil. All samples were stored in refrigerator until transit to the laboratory for analysis.

Full details of sample collection points are summarized in Table S2.

Table S2: Details of sample collection points.

<table>
<tr><th>Event</th><th>Location</th><th colspan="3">Surface Areas:
hands 78 cm^2, throat 60 cm^2, jaws 75 cm^2, and neck 60 cm^2. SCBA 45 cm^2, zipflap 75 cm^2, shoulder 75 cm^2, gloves 100 cm^2, hood 60 cm^2, general office and PPE room 70 cm^2, fire engine door and console 70 cm^2, bodyguard 100 cm^2</th></tr>
<tr><td>Training</td><td>One container</td><td>No of subjects
FF1: Instructor
FF2-4: Trainees</td><td>Skin samples
Neck
Hands
Throat
Jawline</td><td>PPE Samples
Exterior of gloves
Zipper cover
Shoulder of jacket
Hood Exterior of BA</td></tr>
<tr><td rowspan="4">Station1</td><td>General Office</td><td colspan="3">Environmental wipe samples
1. Desk in middle of room; 2. Top of letterbox; 3. Desk next to corridor
Environmental air samples
XAD tube connected to a pump with 1 L/min for 6 hours</td></tr>
<tr><td>PPE Room</td><td colspan="3">Environmental wipe samples
1. Top wall corner; 2. Corner between boot lockers; 3. Wall behind jackets
Environmental air samples
1 XAD tube connected to a pump on a boot locker, the other XAD tube connected to a second pump on a helmet shelf, both sampled at 1 L/min for 6 hours</td></tr>
<tr><td>Fire engine 1</td><td colspan="3">Environmental wipe samples
Door, bodyguard and console wiped 3 times: before, 1 day and 5 days after the cinema fire
Environmental air samples
Collected once: 5 days after the cinema fire, XAD tube connected to a pump with 1 L/min for 4 hours</td></tr>
<tr><td>Fire engine 2</td><td colspan="3">Environmental wipe samples
Door, bodyguard and console wiped 3 times: before, 1 day and 5</td></tr>
</table>

		days after the cinema fire<br>**Environmental air samples**<br>Collected twice: 1 and 5 days after the cinema fire, 1 XAD tube connected to a pump with 1 L/min for 4 hours
Station 2	General Office	**Environmental wipe samples**<br>1.Windows; 2. Desk in middle of the room; 3. Shelf at head-height<br>**Environmental air samples**<br>1 XAD tube connected to a pump with 1 L/min for 6 hours
	Appliance Bay	**Environmental wipe samples**<br>1. Top wall corner; 2. Corner between boot lockers; 3.Wall behind jackets<br>**Environmental air samples**<br>1 XAD tube connected to a pump on a boot locker, the other XAD tube connected to a second pump on a helmet shelf, both sampled at 1 L/min for 6 hours

Supplemental Material 3

Sampling analysis

For the sample analysis, filter and resins from the XAD tubes were placed in individual vials to which 2 mL of hexane/acetone (3:1 v/v) was added. Samples were sonicated for 45 minutes prior to GC/MS analysis.

Wipe samples were sonicated for 30 minutes and then 1.0 mL of each sample was centrifuged for 30 minutes at 13.000 rpm. The solvent was then transferred to amber glass vials and analysed by GC/MS.

Quantitative analysis was performed for PAHs, listed in Table S3, using a HP 6890 Series GC System equipped with a 5973 Mass Selective Detector (Hewlett Packard) and a TraceGOLD TG-5MS column with the dimensions 30 m x 0.25 mm x 0.1 um (Thermo Scientific). An injection volume of 2µl was used with a splitless injection with a flow rate of 35 mL/min.

Samples were analysed in SIM mode. The GC was set to a start temperature of 100°C with a 2 minute hold, then with 8°C/min to 210°C, with 2°C/min to 280°C and was held at 280°C for 3 minutes. The results were then processed using Agilent MSD Chemstation version F.01.00.1903.

Table S3 List of PAHs and the ions scanned for during quantitative analysis

**Compound**	**Molecular Weight**	**Primary Ion**	**Secondary Ion**
Naphthalene	128.2	128	
Benzo(a)anthracene	228.3	228	114
Chrysene	228.3	228	114
Benzo(b)fluoranthene	252.3	252	126
Benzo(k)fluoranthene	252.3	252	126
Benzo(a)pyrene	252.3	252	126
Dibenz(a,h)anthracene	278.3	278	139
7,12-dimethylbenz(a)anthracene	252	126	252
3-methylcholanthrene	268	252	126
Dibenzo(a,e)pyrene	302	302	151

Benzo(j)fluoranthene	252	126	252

Supplemental Material 4

Table S4 Firefighters questionnaires

	**Training in a container**			
Subject	Instructor	Trainee	Trainee	Trainee
FF age	45	47	40	38
Gender	M	M	M	M
Smoker Y/N	N	N	N	Y
Occupation [full time (FT) / part time (PT)]	FF (FT)	FF (FT)	FF (FT)	Mechanic (FT)
Time as a firefighter (years)	22	24	2.5	3
Have you been barbecuing or grilling food in the last week? Y/N	N	N	Y	N
Yes, I've operated a barbecue or grill myself (days since)				
Yes, but I did not operate one myself (days since)			8	
Time since last fire exposure:	24 h	15 h	3 weeks	2 weeks
Position/distance to the fire	next to it – 1.50m	3m outside	hose, 10m	
Exposed to: (fume, soot, smoke, heat)	fume, soot, steam	fume, soot	fume, soot	fume, soot
Smoke Diving Time:	1 hour			1 hour
Total time at the fire site: (hours)	6	2	1	3
Time gear was worn during task (hours):	6	2	1	3
Time since gear was last cleaned: (days)	6	60	14	28
Times gear has been used since last cleaning:	1	10	3	0
Age of gear worn during study (years)	10	8	2.5	3

References

Agency for Toxic Substances and Disease Registry. 2017. Minimal Risk Levels June 2017. https://www.atsdr.cdc.gov/mrls/pdfs/atsdr_mrls.pdf [Accessed 17 July 2017].

IARC (International Agency for Research on Cancer). 2010. Some Non-heterocyclic Polycyclic Aromatic Hydrocarbons and Some Related Exposures. IARC Monogr Eval Carcinog Risk Hum 92:754-774.

Office of Environmental Health Hazard Assessment (OEHHA). 1992. Expedited cancer Potency values and Proposed regulatory Levels for certain Proposition 65 Carcinogens. Available: https://oehha.ca.gov/media/downloads/proposition-65/report/expcancer.pdf [Accessed 17 July 2017].

Office of Environmental Health Hazard Assessment (OEHHA). 2011. Appendix A lookup table with 2011 approved TEFs. Available: https://oehha.ca.gov/media/downloads/crnr/appendixa.pdf [Accessed 17 July 2017].

Office of Environmental Health Hazard Assessment (OEHHA). 2017. Air Chemicals Database. Available: https://oehha.ca.gov/air/chemicals [Accessed 17 July 2017].

U.S. EPA. 1988. Evaluation of the Potential Carcinogenicity of 7,12-Dimethylbenz(a)anthracene (57-97-6). Research Triangle Park, NC:U.S. Environmental Protection Agency.

U.S. EPA. 1990. Dibenz[a,h]anthracene (CASRN 53-70-3) | IRIS | US EPA. Available: https://cfpub.epa.gov/ncea/iris/iris_documents/documents/subst/0456_summary.pdf [Accessed 17 July 2017].
